# A Self‐Assembling *β*‐Annulus Nanoparticle Vaccine Incorporating OX40L Confers Partial Protection Against NADC30‐Like PRRSV in Piglets

**DOI:** 10.1155/tbed/2092704

**Published:** 2026-10-08

**Authors:** Linxing Tian, Zhengdan Lin, Jiahui Ma, Zihui Hu, Nan Cao, Xinghua Chen, Mingxing Lu, Shudan Liu, Chao Zhang, Huanchun Chen, Xiangmin Li, Ping Qian

**Affiliations:** ^1^ National Key Laboratory of Agricultural Microbiology Hubei Hongshan Laboratory Huazhong Agricultural University Wuhan 430070 Hubei China hzau.edu.cn; ^2^ College of Veterinary Medicine Huazhong Agricultural University Wuhan 430070 Hubei China hzau.edu.cn; ^3^ Key Laboratory of Preventive Veterinary Medicine in Hubei Province The Cooperative Innovation Center for Sustainable Pig Production Wuhan 430070 Hubei China; ^4^ Hubei Jiangxia Laboratory Wuhan 430200 Hubei China

**Keywords:** immunogenicity, NADC30-like strains, nanoparticle vaccine, protective efficacy, PRRSV, *β*-annulus peptide

## Abstract

Porcine reproductive and respiratory syndrome (PRRS) remains one of the most economically significant viral diseases affecting the global swine industry, and currently available commercial vaccines provide only limited protection against circulating PRRS virus (PRRSV) strains. Therefore, alternative vaccination strategies are urgently needed to enhance protection against PRRSV infection. In this study, selected antigenic regions derived from the PRRSV structural proteins GP3, GP4, GP5, and M were incorporated into a *β*‐annulus peptide‐based nanoparticle scaffold derived from tomato bushy stunt virus (TBSV), and the resulting construct was further fused with the immunostimulatory molecule OX40L. The resulting OX40L‐*β*‐MEs protein formed nanoscale particles and was evaluated for immunogenicity and protective efficacy in piglets. Immunization with the OX40L‐*β*‐MEs nanoparticle vaccine induced measurable PRRSV‐specific humoral responses and cellular immune‐related responses in piglets. Following the challenge with the NADC30‐like PRRSV strain GX‐3264, piglets in the OX40L‐*β*‐MEs group showed shorter fever duration, reduced serum viremia and lung viral loads, and milder pulmonary lesions compared with PBS controls. In summary, these findings demonstrate that the *β*‐annulus‐based nanoparticle formulation can induce measurable immune responses and provide partial protection against NADC30‐like PRRSV challenge, supporting further evaluation of this strategy for PRRSV vaccine development.

## 1. Introduction

Porcine reproductive and respiratory syndrome (PRRS) is a viral disease caused by the PRRS virus (PRRSV), which affects both the respiratory and reproductive systems, leading to substantial economic losses to the global swine industry annually [1, 2]. PRRSV is an enveloped, single‐stranded positive‐sense RNA virus belonging to the genus *Betaarterivirus*, family Arteriviridae, and order Nidovirales [3]. The PRRSV genome is ~15.4 kb in length and encodes eight structural proteins, including four membrane‐associated glycoproteins (GP2, GP3, GP4, and GP5), three nonglycosylated membrane proteins (E, ORF5a, and M), and the nucleocapsid protein (N) [4].

The high mutation rate and frequent recombination of PRRSV have resulted in the emergence of multiple recombinant strains, presenting a considerable challenge for vaccine development [5]. Currently, commercial PRRSV vaccines primarily include inactivated and live‐attenuated vaccines. Inactivated vaccines generally offer limited protection, whereas live attenuated vaccines are primarily effective against homologous strains and may also carry a risk of reversion to virulence [6, 7]. In China, where NADC30‐like PRRSV has become the predominant circulating strain and multiple recombinant lineages cocirculate, the protective efficacy of existing commercial vaccines remains limited [8, 9].

Although multiple PRRSV structural proteins collectively contribute to protective immunity, the complex oligomerization and extensive glycosylation of viral surface proteins may interfere with neutralizing antibody recognition [10]. Therefore, novel design strategies are required to enhance vaccine immunogenicity. Nanoparticle scaffolds have emerged as promising antigen delivery platforms for vaccine development [11, 12]. Compared with soluble protein subunits, nanoparticle scaffolds can present antigens in a multivalent manner on their surface, thereby facilitating uptake by antigen‐presenting cells (APCs) and enhancing immunogenicity and structural stability [12]. The *β*‐annulus peptide derived from plant viruses, such as tomato bushy stunt virus (TBSV), can self‐assemble into nanocapsular structures, highlighting its potential as a versatile platform for vaccine development [13].

Both humoral and cellular immune responses are critical for clearing PRRSV infection [14, 15]. Compared with live‐attenuated PRRSV vaccines, inactivated vaccines generally induce weaker cellular immune responses, which may partly account for their suboptimal protective efficacy [16]. Therefore, enhancing vaccine‐induced cellular immunity has become a key objective in the development of next‐generation PRRSV vaccines [6]. Previous studies have shown that codelivery of mRNA encoding the T‐cell costimulatory molecule OX40L, compared with cytokine mRNA alone, significantly improves complete response rates in distal tumors [17]. Similarly, coadministration of OX40L with a foot‐and‐mouth disease virus (FMDV) VP1‐based DNA vaccine was reported to enhance both humoral and cellular immune responses and to improve vaccine‐mediated protection, suggesting that OX40L has the potential to augment vaccine immunogenicity [18].

In this study, we designed a recombinant monomeric antigen protein (MEs) comprising selected antigenic regions derived from PRRSV structural proteins GP3, GP4, GP5, and M. MEs was subsequently fused with the *β*‐annulus peptide to generate *β*‐MEs, which was further fused with OX40L to generate OX40L‐*β*‐MEs. Animal experiments were performed to evaluate the immunogenicity and protective efficacy of these nanoparticle formulations against NADC30‐like PRRSV challenge in piglets.

## 2. Materials and Methods

### 2.1. Cells, Viruses, and Materials

NADC30‐like PRRSV strain GX‐3264 (GenBank Accession No. PV240231) was stored in our laboratory. Porcine alveolar macrophages (PAMs) were isolated from healthy 4‐week‐old piglets. The cells were cultured in RPMI‐1640 medium (Gibco, Waltham, MA, USA) supplemented with 10% fetal bovine serum (FBS), penicillin (100 U/mL), and streptomycin (100 μg/mL) at 37°C in a humidified incubator containing 5% CO_2_. The prokaryotic expression vector pCold II was maintained in our laboratory.

### 2.2. Plasmid Construction

The recombinant monomeric antigen construct (MEs) was designed based on the PRRSV strain GX‐3264 (GenBank Accession No. PV240231). MEs comprised selected antigenic regions derived from the PRRSV structural proteins GP3, GP4, GP5, and M.

The amino acid positions, lengths, sequences, and selection rationale for each antigenic region are provided in Table S1. The *β*‐annulus peptide gene sequence was derived from TBSV (GenBank Accession No. U80935.1), and the OX40L gene sequence was obtained from *Sus scrofa* tumor necrosis factor superfamily member 4 (TNFSF4) (GenBank Accession No. NM_001025217). The corresponding DNA fragments were generated by overlap PCR and subsequently cloned into the pCold II expression vector, yielding the recombinant plasmids pCold Ⅱ‐MEs, pCold Ⅱ‐*β*‐MEs, and pCold Ⅱ‐OX40L‐*β*‐MEs.

### 2.3. Protein Expression and Purification

The three recombinant plasmids (pCold Ⅱ‐MEs, pCold Ⅱ‐*β*‐MEs, and pCold Ⅱ‐OX40L‐*β*‐MEs) were individually transformed into *E. coli* BL21 cells harboring the pG‐Tf2 molecular chaperone plasmid. Bacterial cultures were grown in an LB medium containing chloramphenicol (20 μg/mL), ampicillin (100 μg/mL), and tetracycline (5 ng/mL) at 37°C with shaking. When the OD_600_ reached 0.6–0.8, isopropyl *β*‐D‐1‐thiogalactopyranoside (IPTG) was added to a final concentration of 0.4 mmol/L to induce protein expression. The cultures were then incubated at 16°C with shaking at 160 rpm for 20 h, and the harvested cells were resuspended in Tris‐HCl buffer (pH 8.0). The cell suspension was disrupted by low‐temperature ultrasonication (JNBIO, Shanghai, China), followed by centrifugation at 12,000 rpm for 20 min at 4°C. The supernatant was filtered through a 0.22 μm membrane and subjected to preliminary purification using the Ni Sepharose 6 Fast Flow resin (GE Healthcare, Shanghai, China). Further purification and analysis were performed by size‐exclusion chromatography (SEC) using a HiLoad 16/600 Superdex 75 prep‐grade column (GE Healthcare, Shanghai, China).

### 2.4. SDS‐PAGE and Western Blotting

The molecular weights of the recombinant proteins were analyzed by SDS‐PAGE and western blotting, as previously described [19]. Protein samples were collected, resuspended in 5× loading buffer, boiled at 95°C for 10 min, and then cooled on ice for 2 min. The samples were then subjected to SDS‐PAGE and western blotting. SDS‐PAGE gels were stained with Coomassie Brilliant Blue (Absin Biology, China) at room temperature for 60 min and subsequently destained until the background became clear. For western blotting, a mouse anti‐His monoclonal antibody (ABClone, China; 1:5000) was used as the primary antibody. HRP‐conjugated goat anti‐mouse IgG (ABClone, China; 1:5000) was used as the secondary antibody. Protein bands were visualized using an enhanced chemiluminescence (ECL) detection system and analyzed using Image Lab software (version 4.0.1; Bio‐Rad, Hercules, CA, USA).

### 2.5. Dynamic Light Scattering (DLS) Detection

DLS measurements were performed using a DAWN HELEOS 18‐angle scatterer (Wyatt, Inc., USA), as previously described [20]. Briefly, purified nanoparticle proteins were filtered through a 0.22 μm membrane. The proteins were diluted to 0.1 mg/mL in Tris‐HCl buffer (pH 8.0). The hydrodynamic diameters of the nanoparticle proteins were measured at 25°C, and the mean values were calculated from 10 independent measurements. Data were analyzed using Dynamics software Version 7.8.1.3.

### 2.6. Bio‐Transmission Electron Microscopy (TEM) Analysis

The morphology of the nanoparticle proteins was evaluated using TEM, as previously described [21]. Briefly, 10 μL of the sample (0.1 mg/mL) was applied onto a carbon‐coated grid that had been glow‐discharged in air for 1 min. The grid was immediately negatively stained with 2% phosphotungstic acid and 2% ammonium molybdate, followed by air drying for 60 s. Images were acquired using a Bio‐TEM instrument (FEI Company, Hillsboro, OR, USA).

### 2.7. Immunization and Challenge of Piglets

Twenty‐five 4‐week‐old piglets negative for PRRSV, ASFV, CSFV, PCV2, and PRV were randomly divided into five groups (*n* = 5): the MEs group, *β*‐MEs group, OX40L‐*β*‐MEs group, Inactivated vaccine group, and PBS control group. The recombinant protein formulations were emulsified with the MONTANIDE ISA 201VG adjuvant at a 1:1 (w/o/w) ratio. Each piglet received 2 mL of vaccine containing 100 μg of protein by an intramuscular injection. Piglets in the inactivated vaccine and PBS groups received the same injection volume (Table S2). A booster immunization was administered at 21 days postvaccination (dpv) using the same dose. Blood samples were collected at 0, 14, 28, and 42 dpv for subsequent analysis. At 42 dpv, piglets were challenged with the NADC30‐like PRRSV strain GX‐3264 at a total dose of 4 × 10^5^ TCID_50_ via combined intranasal and intramuscular routes. Rectal temperature, clinical signs, and body weight were monitored after the challenge. Clinical scoring criteria were applied as previously described [22]. Blood samples were collected at 0, 3, 5, 7, 10, and 14 days postinfection (dpi) to evaluate viremia. At 14 dpi, piglets were euthanized for necropsy, and lung tissues were collected for viral load quantification, histopathology, and IHC analysis.

### 2.8. Enzyme‐Linked Immunosorbent Assay (ELISA)

PRRSV‐specific IgG antibodies were measured using an indirect ELISA, as previously described [23]. Briefly, plates were coated overnight at 4°C with purified inactivated PRRSV GX‐3264 (0.5 μg/well). The plates were then blocked with 2% bovine serum albumin (BSA) at 37°C for 2 h. After washing three times with PBST, 100 μL of serum diluted 1:3200 was added to each well and incubated at 37°C for 1 h. After three washes with PBST, HRP‐conjugated goat anti‐pig IgG (H + L) antibody (1:10,000; ABClone, China) was added and incubated at 37°C for 1 h. After washing three times with PBST, 100 μL of TMB substrate solution (Solarbio, Beijing, China) was added and incubated at 37°C for 15 min. The reaction was stopped using a stop solution (Solarbio, Beijing, China), and the absorbance was measured at 450 nm using a multimode plate reader.

### 2.9. Virus Neutralization (VN) Assay

VN assays were conducted in PAMs based on a previously described protocol [24]. Briefly, serum samples were heat‐inactivated at 56°C for 30 min and then serially two‐fold diluted in RPMI‐1640 medium. Equal volumes of diluted serum and PRRSV GX‐3264 (100 TCID_50_) were mixed and incubated at 37°C for 1 h. Subsequently, 100 μL of PAM cell suspension was added to each well, and the plates were incubated at 37°C for 48 h. Cells were then fixed and subjected to an indirect immunofluorescence assay (IFA) using an anti‐PRRSV N protein antibody. PRRSV‐infected cells were identified by fluorescent foci. The neutralizing antibody titer was defined as the highest serum dilution that reduced PRRSV infection by 50%.

### 2.10. Lymphocyte Proliferation Assay

At 42 dpv, PBMC proliferation after PRRSV restimulation was evaluated using a CCK‐8 assay, as previously described [25]. Briefly, peripheral blood mononuclear cells (PBMCs) were isolated from piglets in each immunization group at 42 dpv. PBMCs were seeded at 5 × 10^6^ cells/mL and restimulated with inactivated PRRSV GX‐3264 (0.1 MOI). After incubation at 37°C for 72 h, 10 μL of CCK‐8 reagent was added to each well and incubated for an additional 4 h at 37°C. Absorbance was measured at 450 nm using a microplate reader. The stimulation index (SI) was calculated as follows: (OD of the immune group − OD of the blank control)/(OD of the negative control group − OD of the blank control).

### 2.11. Cytokine Assays

For cytokine analysis, PBMCs isolated at 42 dpv were restimulated with inactivated PRRSV GX‐3264 (0.1 MOI) at 37°C for 48 h. Culture supernatants were then collected, and the concentrations of IL‐2, IL‐10, and IFN‐γ were measured using porcine cytokine ELISA kits (NeoBioscience, Wuhan, China) according to the manufacturer’s instructions. Absorbance was measured at 450 nm using a microplate reader, and cytokine concentrations were calculated based on the standard curves.

### 2.12. Flow Cytometry Analysis

The proportions of CD3^+^, CD4α^+^, and CD8α^+^ lymphocytes following in vitro PRRSV restimulation were analyzed by flow cytometry, as described previously [26]. Briefly, the isolated PBMCs were stimulated with inactivated PRRSV GX‐3264 (0.1 MOI). Concanavalin A (ConA) was used as a positive control. After incubation at 37°C for 10 h, the GolgiPlug reagent (BD Biosciences, USA) was added to each well, and the cells were incubated for an additional 4 h at 37°C. Cells were then harvested and stained with the following antibodies according to the manufacturer’s instructions: FITC‐conjugated mouse anti‐pig CD3ε (clone BB23‐8E6‐8C8), Alexa Fluor 647‐conjugated mouse anti‐pig CD4α (clone 74‐12‐4), and PE‐conjugated mouse anti‐pig CD8α (clone 76‐2‐11, BD Biosciences, USA). Flow cytometric data were acquired using a flow cytometer (CytoFLEX, Beckman Coulter, Pasadena, CA, USA) and analyzed using CytExpert software (Version 2.2; Beckman Coulter, Pasadena, CA, USA).

### 2.13. Quantitative Reverse Transcription PCR (RT‐qPCR)

PRRSV RNA loads in serum and lung tissues were quantified by RT‐qPCR, as previously described [27]. Briefly, RNA was extracted from the samples using the E.Z.N.A. Viral RNA Kit (Omega Bio‐Tek, USA) was used according to the manufacturer’s instructions. RT‐qPCR was performed using the HiScript II U + One Step RT‐PCR Probe Kit (Vazyme, Nanjing, China) on a ViiA 7 Real‐Time PCR system (Applied Biosystems, Grand Island, NY, USA). The primer sequences used in this study are listed in Table S3. A standard curve was generated using 10‐fold serial dilutions of a recombinant plasmid containing the PRRSV ORF6 gene.

### 2.14. Histopathology and Immunohistochemistry

Lung tissue lesions and PRRSV N protein‐positive signals were evaluated by H&E staining and IHC, respectively, as previously described [28]. Briefly, tissue samples were fixed in 4% paraformaldehyde and embedded in paraffin. Tissue sections were then subjected to H&E staining, and IHC was performed using a rabbit anti‐PRRSV N protein antibody (GeneTex, Inc., Irvine, CA, USA) according to the manufacturer’s instructions. PRRSV N protein‐positive staining was quantified using ImageJ software (Version 1.54 g, National Institutes of Health, USA). Five nonoverlapping lung fields were analyzed per animal, and the mean percentage of the positive staining area was used for statistical analysis.

### 2.15. Statistical Analysis

Statistical analyses were performed using GraphPad Prism 8.0.2 (GraphPad Software, San Diego, CA, USA). Data are presented as the mean ± standard deviation (SD). Comparisons were conducted using one‐way or two‐way ANOVA, followed by Tukey’s test. For nonparametric comparisons, the Kruskal–Wallis test followed by the Dunn’s multiple‐comparison test was used. A *p* value of <0.05 was considered to indicate statistical significance.

## 3. Results

### 3.1. Construction and Expression of Recombinant Proteins

Multiple PRRSV structural proteins have been reported to contribute to protective immunity against PRRSV, and OX40L has been shown to enhance the immunogenicity of fusion proteins as a costimulatory molecule. Therefore, based on the ability of the *β*‐annulus peptide to serve as a self‐assembling nanoparticle scaffold for surface antigen display, three recombinant proteins (MEs, *β*‐MEs, and OX40L‐*β*‐MEs) were designed and constructed (Figure 1A). The *β*‐annulus peptide has been reported to self‐assemble in vitro into nanocapsular structures. Based on this property, *β*‐MEs and OX40L‐*β*‐MEs were designed to form self‐assembling nanoparticle structures (Figure 1B, C). Recombinant MEs, *β*‐MEs, and OX40L‐*β*‐MEs proteins were expressed by *E. coli* BL21 and subsequently purified by Ni‐affinity chromatography. The purified proteins were analyzed by SDS‐PAGE and western blotting, revealing molecular weights of ~32, 36, and 39 kDa for MEs, *β*‐MEs, and OX40L‐*β*‐MEs, respectively (Figure 1D).

**Figure 1:**
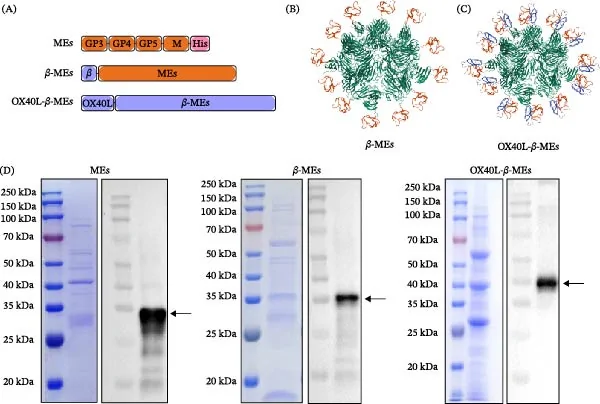
Construction and expression of recombinant proteins. (A) Schematic diagram of the recombinant protein constructs MEs, *β*‐MEs, and OX40L‐*β*‐MEs. Schematic models of the proposed *β*‐MEs (B) and OX40L‐*β*‐MEs (C) assemblies. (D) Analysis of recombinant proteins by SDS‐PAGE (left) and western blotting (right).

### 3.2. Characterization of Nanoparticles

The recombinant proteins were further analyzed and purified by SEC. The SEC profiles exhibited distinct elution peaks for both *β*‐MEs and OX40L‐*β*‐MEs, and the corresponding fractions were collected for subsequent SDS‐PAGE analysis (Figure 2A, D). SDS‐PAGE results revealed protein bands consistent with the expected molecular weights. To assess the nanoparticle‐forming capability of the *β*‐annulus peptide, the size and morphology of the purified proteins were further analyzed by DLS and TEM. DLS analysis indicated that the average hydrodynamic diameters of *β*‐MEs and OX40L‐*β*‐MEs measured at 25°C were ~25.85 nm and 41.57 nm, respectively (Figure 2B, E). Consistent with the DLS results, TEM analysis confirmed that *β*‐MEs and OX40L‐*β*‐MEs assembled into spherical nanoparticles (Figure 2C, F). Collectively, these results demonstrate that *β*‐MEs and OX40L‐*β*‐MEs self‐assemble into nanoparticles.

**Figure 2:**
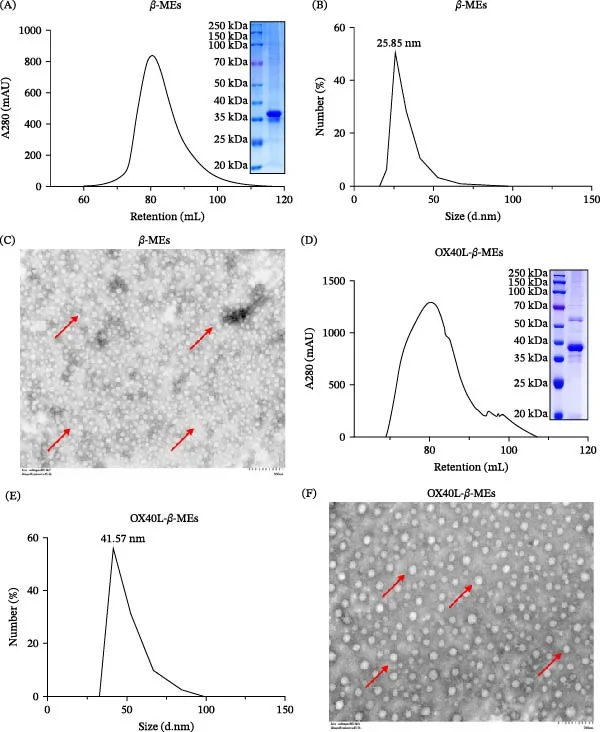
Characterization of self‐assembled nanoparticle proteins. Purification of *β*‐MEs (A) and OX40L‐*β*‐MEs (D) from *E. coli* (BL21) using size‐exclusion chromatography (SEC) and SDS‐PAGE analysis. Hydrodynamic diameters of the *β*‐MEs (B) and OX40L‐*β*‐MEs (E) analyzed by dynamic light scattering (DLS) at 25°C. Negative‐staining TEM images of *β*‐MEs (C) and OX40L‐*β*‐MEs (F) nanoparticles, with red arrows indicating the particles.

### 3.3. PRRSV Nanoparticle Vaccines Induce Humoral Immune Responses

To evaluate the humoral immune responses induced by the nanoparticle vaccines in piglets, animals were immunized with MEs, *β*‐MEs, OX40L‐*β*‐MEs, the commercial inactivated vaccine, or PBS at 0 and 21 dpv (Figure 3A). Serum samples were collected at 0, 14, 28, and 42 dpv to measure PRRSV‐specific IgG antibodies. PRRSV‐specific IgG antibodies were gradually induced in all vaccinated groups after immunization. At 14 dpv, the inactivated vaccine induced significantly higher PRRSV‐specific IgG levels than the other vaccinated groups (*p* < 0.05), whereas IgG levels in the MEs, *β*‐MEs, and OX40L‐*β*‐MEs groups remained relatively low and were not significantly different from those in the PBS group (*p* > 0.05). At 28 dpv, PRRSV‐specific IgG levels in the *β*‐MEs and OX40L‐*β*‐MEs groups gradually increased but remained significantly lower than those in the inactivated vaccine group, with no significant difference compared with the PBS group (*p* > 0.05). By 42 dpv, PRRSV‐specific IgG levels in both nanoparticle vaccine groups had further increased and reached levels comparable to those induced by the inactivated vaccine (Figure 3B, *p* > 0.05). In addition, virus‐neutralizing antibody titers were evaluated at the same time points. At 42 dpv, low levels of neutralizing antibodies were detected in all vaccinated groups, with no significant differences among the groups (Figure 3C, *p* > 0.05). Collectively, these results indicate that the *β*‐annulus‐based nanoparticle formulations elicited measurable PRRSV‐specific humoral immune responses, mainly reflected by the induction of IgG antibodies after booster immunization, although neutralizing antibody responses remained limited.

**Figure 3:**
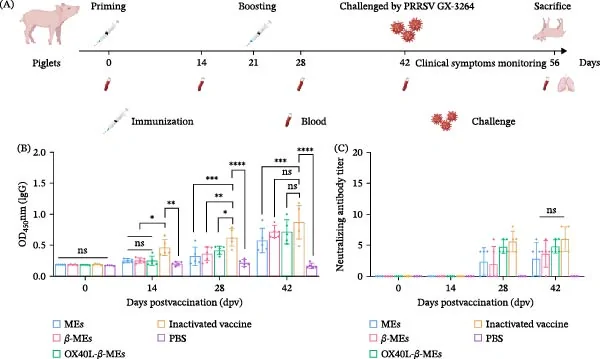
Humoral immune responses induced by PRRSV nanoparticle vaccines in piglets. (A) Schematic representation of the immunization and challenge study design. (B) PRRSV‐specific IgG levels in serum samples collected at 0, 14, 28, and 42 dpv were determined by indirect ELISA. (C) PRRSV neutralizing antibody titers in serum at different time points. Data are presented as mean ± SD. Significance was determined by one‐way or two‐way ANOVA ( ^∗^, *p* < 0.05;  ^∗∗^, *p* < 0.01;  ^∗∗∗^, *p* < 0.001;  ^∗∗∗∗^, *p* < 0.0001; ns, no significant difference).

### 3.4. PRRSV Nanoparticle Vaccines Induce Cellular Immune Responses

To evaluate vaccine‐induced cellular immune responses, PBMCs were isolated from piglets at 42 dpv and stimulated with inactivated PRRSV GX‐3264. Flow cytometry was performed to determine the proportions of CD3^+^, CD4α^+^, and CD8α^+^ lymphocytes among PBMCs from the different experimental groups. In the OX40L‐*β*‐MEs group, the proportions of CD3^+^, CD4α^+^, and CD8α^+^ lymphocytes were 32.84%, 16.33%, and 41.21%, respectively. The percentages of CD3^+^, CD4α^+^, and CD8α^+^ lymphocytes in the OX40L‐*β*‐MEs group were significantly higher than those in the PBS control group (*p* < 0.0001). Moreover, the OX40L‐*β*‐MEs nanoparticle vaccine group exhibited significantly higher CD8α^+^ lymphocyte proportions than the other vaccine groups (Figure 4A and Figure S1, *p* < 0.0001). Lymphocyte proliferation assays revealed that the OX40L‐*β*‐MEs group exhibited a significantly higher SI (SI = 2.65) compared with the PBS control group (SI = 0.99) (Figure 4B, *p* < 0.0001). Furthermore, cytokine production by PRRSV‐stimulated PBMCs was quantified using an ELISA. The levels of IL‐2 (*p* < 0.0001), IL‐10 (*p* < 0.0001), and IFN‐γ (*p* < 0.01) secreted by PBMCs from the OX40L‐*β*‐MEs group were significantly elevated compared with those from the PBS control group (Figure 4C–E). Collectively, these results indicate that the OX40L‐*β*‐MEs nanoparticle vaccine elicited measurable cellular immune‐related responses following PRRSV restimulation in piglets.

**Figure 4:**
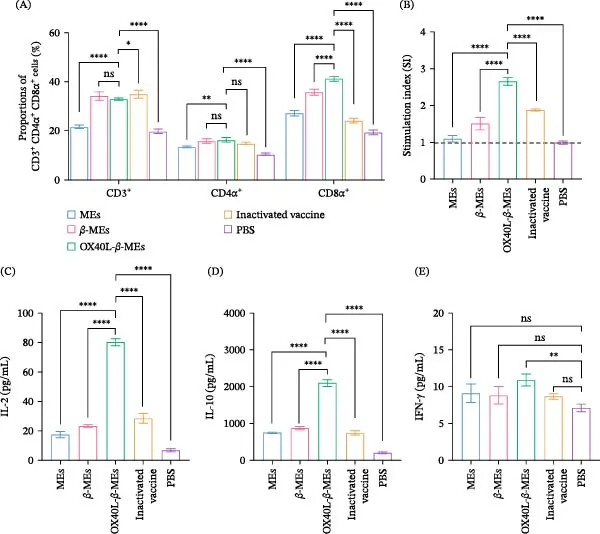
Cellular immune responses induced by PRRSV nanoparticle vaccines in piglets. (A) The proportions of CD3^+^, CD4α^+^, and CD8α^+^ lymphocytes in PBMCs following in vitro PRRSV restimulation were analyzed by flow cytometry. (B) Proliferative responses of PBMCs following PRRSV stimulation. (C–E) The concentrations of IL‐2 (C), IL‐10 (D), and IFN‐γ (E) in the culture supernatants of PRRSV‐restimulated PBMCs were measured using commercial ELISA kits. Data are presented as mean ± SD. Significance was determined by one‐way or two‐way ANOVA ( ^∗^, *p* < 0.05;  ^∗∗^, *p* < 0.01;  ^∗∗∗^, *p* < 0.001;  ^∗∗∗∗^, *p* < 0.0001; ns, no significant difference).

### 3.5. PRRSV Nanoparticle Vaccines Confer Partial Protection Against NADC30‐Like PRRSV Challenge in Piglets

To evaluate the protective effects of PRRSV nanoparticle vaccines, all piglets were challenged with the NADC30‐like PRRSV strain GX‐3264 at 42 dpv. After the challenge, rectal temperature, body weight, and clinical signs were monitored daily in all piglets. Piglets were euthanized at 14 dpi, and lung tissues were collected for histopathological examination and viral load determination. The PBS group exhibited persistent fever (≥40°C) until 10 dpi. In contrast, piglets immunized with OX40L‐*β*‐MEs showed only a transient increase in rectal temperature and subsequently returned to the normal range (Figure 5A). During the challenge period, ADWG in the PBS control group was markedly reduced, consistent with their more severe clinical signs, and was significantly lower than that in the OX40L‐*β*‐MEs group (Figure 5B, *p* < 0.01). Consistent with these observations, all vaccinated groups showed alleviated clinical signs compared with the PBS control group. Among the vaccinated groups, piglets in the OX40L‐*β*‐MEs group showed a relatively mild clinical presentation (Figure 5E). These results demonstrate that the OX40L‐*β*‐MEs nanoparticle vaccine alleviated clinical symptoms in PRRSV‐challenged piglets.

**Figure 5:**
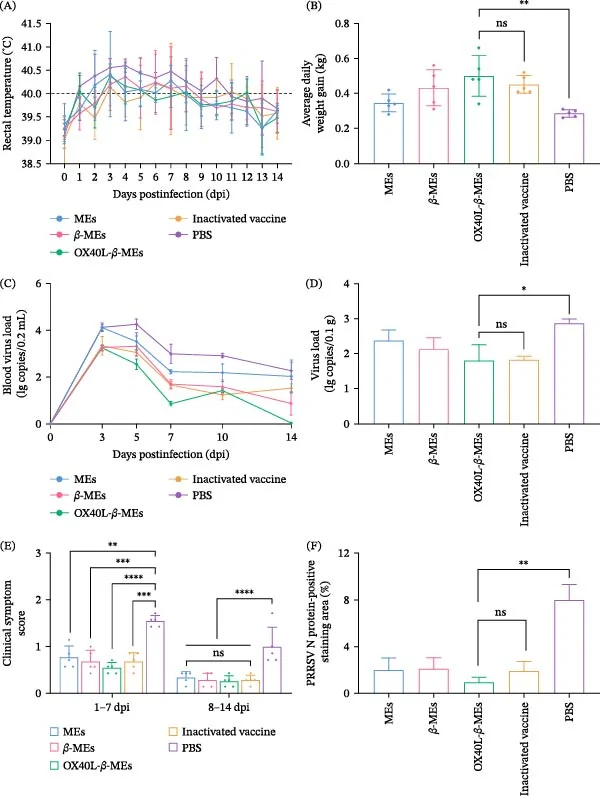
Protective effects of PRRSV nanoparticle vaccines against NADC30‐like PRRSV challenge in piglets. (A) Rectal temperature of piglets following challenge. Piglets were challenged with the NADC30‐like PRRSV strain GX‐3264 at 42 dpv, and rectal temperatures were recorded daily. Fever was defined as a rectal temperature ≥40°C. (B) Average daily weight gain (ADWG) of piglets following challenge. (C) Viral loads in serum samples collected at different time points after PRRSV challenge were determined by RT‐qPCR. (D) Viral loads in the lung tissues of piglets were determined. (E) After challenge, clinical symptoms were monitored and scored daily. Mental state, respiration, and coughing were observed, with each scored on a scale of 0–4: 0 for normal, 1 for very mild symptoms, 2 for mild symptoms, 3 for severe symptoms, and 4 for death. Totals and mean values were calculated for each group. (F) Quantification of PRRSV N protein‐positive staining area in lung tissues. Data are presented as mean ± SD. Significance was analyzed using the Kruskal–Wallis test followed by Dunn’s multiple‐comparison test or two‐way ANOVA ( ^∗^, *p* < 0.05;  ^∗∗^, *p* < 0.01;  ^∗∗∗^, *p* < 0.001;  ^∗∗∗∗^, *p* < 0.0001; ns, no significant difference).

Additionally, serum viral loads were monitored by RT‐qPCR throughout the challenge period. The results showed that although viremia was detected in all piglets at 3 dpi, the *β*‐MEs, OX40L‐*β*‐MEs, and inactivated vaccine groups showed lower serum viral loads than the PBS group during the challenge period. Notably, the OX40L‐*β*‐MEs vaccine group showed relatively low levels of viremia throughout the challenge period, with viral RNA falling below the detection limit in serum at 14 dpi (Figure 5C). Similarly, the OX40L‐*β*‐MEs vaccine group showed significantly lower lung viral loads than the PBS group (*p* < 0.05), whereas no significant difference was observed between the OX40L‐*β*‐MEs and inactivated vaccine groups (Figure 5D, *p* > 0.05). These findings suggest that OX40L‐*β*‐MEs vaccination contributed to reduced viral replication in serum and lung tissues after challenge. Gross and microscopic lung lesions were evaluated at 14 dpi. Piglets in the PBS group showed evident pulmonary consolidation and hemorrhagic lesions, whereas vaccinated piglets exhibited milder gross lesions (Figure 6A). Piglets in the MEs group exhibited only mild pulmonary consolidation, whereas inconspicuous gross lesions were observed in the *β*‐MEs, OX40L‐*β*‐MEs, and inactivated vaccine groups (Figure 6A). Consistently, histopathological analysis revealed similar trends, with the PBS group showing typical interstitial pneumonia‐like lesions, including marked alveolar septal thickening, inflammatory cell infiltration, and structural damage. In contrast, lung tissues from the OX40L‐*β*‐MEs nanoparticle vaccine group exhibited only mild lesions, with limited interstitial histopathological changes (Figure 6B). Subsequently, IHC analysis was performed using a PRRSV N protein‐specific antibody to evaluate the PRRSV antigen in lung tissues. The results showed strong PRRSV‐positive signals in lung tissues from the PBS group, whereas only weak PRRSV‐positive signals were detected in the OX40L‐*β*‐MEs and inactivated vaccine groups (Figure 6C). Quantitative analysis further showed that the PRRSV N protein‐positive staining area was significantly lower in the OX40L‐*β*‐MEs group than in the PBS group (*p* < 0.01), whereas no significant difference was observed between the OX40L‐*β*‐MEs and inactivated vaccine groups (Figure 5F, *p* > 0.05). Collectively, these findings indicate that OX40L‐*β*‐MEs provided partial protection against the NADC30‐like PRRSV challenge in piglets.

**Figure 6:**
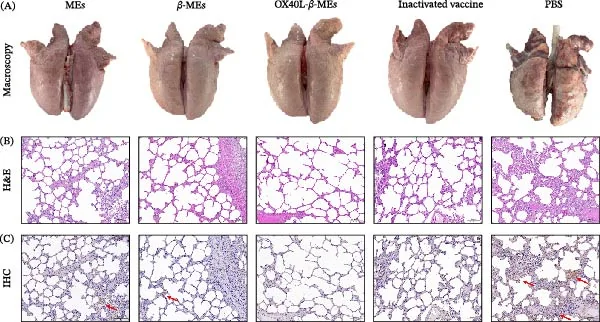
Gross and microscopic pulmonary lesions in piglets after NADC30‐like PRRSV challenge. (A) Gross lung lesions observed at necropsy at 14 dpi. (B) H&E‐stained lung sections showing pulmonary histopathological lesions. Scale bars, 100 μm. (C) Immunohistochemical staining of PRRSV N protein in lung tissues. Brown staining indicates PRRSV N protein‐positive signals. Scale bars, 100 μm.

## 4. Discussion

PRRS is a highly contagious viral disease that has caused substantial economic losses to the global swine industry since its emergence. Although antiviral agents and gene‐edited pigs resistant to PRRSV infection have been developed, vaccination remains the most practical and scalable strategy for the prevention and control of PRRSV transmission [29, 30]. An effective vaccine design requires consideration of the antigenic properties of viral structural proteins and strategies to enhance immunogenicity.

Previous studies have identified glycoprotein GP5 as a major protective antigen of PRRSV [31]. However, the presence of a nonneutralizing decoy epitope (epitope A) and N‐linked glycosylation sites surrounding neutralizing epitope B contribute to glycan shielding, thereby impairing the efficient induction of neutralizing antibodies [32, 33]. Roques et al. [34] demonstrated that modification of the decoy epitope and removal of N‐glycosylation sites in GP5 (M‐GP5m) significantly reduced viremia in infected piglets. Nevertheless, accumulating evidence suggests that GP5 alone is insufficient to confer broad and durable protection and that other structural proteins, including GP3, GP4, and M, also contribute to protective immune responses against PRRSV [35]. These findings highlight the essential roles of multiple structural antigens in the rational design of next‐generation PRRSV vaccines.

Low‐molecular‐weight proteins or small antigens generally exhibit poor immunogenicity and therefore require suitable carriers to ensure effective antigen presentation [12, 36]. Geng et al. expressed Fc‐tagged SARS‐CoV‐2 RBD and protein A‐tagged lumazine synthase (LS), which efficiently assembled in vitro into virus‐like particle (VLP) structures. Compared with soluble RBD, the VLP‐RBD vaccine induced more than a five‐fold increase in neutralizing antibody titers and conferred effective protection against the SARS‐CoV‐2 challenge in mice [37]. Similarly, Chen et al. [38] developed a versatile nanoparticle antigen‐display platform based on PCV2 VLPs, which efficiently presented diverse antigens, including PRRSV B‐cell epitopes, PEDV COE monomers, CSFV E2 dimers, and SIV HA trimers, thereby enhancing Th2‐biased immune responses while also improving Th1 responses and ultimately promoting both humoral and cellular immune responses. In addition, Cao et al. [39] utilized the self‐assembly property of the *β*‐annulus peptide to form nanoparticles conjugated with SVA epitopes, thereby generating a nanoparticle vaccine that effectively protected pigs against SVA infection. Collectively, these studies demonstrate that coupling antigens to nanoparticle scaffolds is an effective strategy to enhance vaccine immunogenicity. However, large scaffold proteins may elicit strong scaffold‐specific immune responses, resulting in immunodominance that may compromise the immunogenicity of displayed antigens. Therefore, the *β*‐annulus peptide, consisting of only 24 amino acids, was selected as the nanoparticle scaffold in this study to minimize scaffold‐induced immunodominance.

In this study, a self‐assembling *β*‐annulus peptide scaffold was fused with selected antigenic regions derived from PRRSV structural proteins to generate a nanoparticle vaccine. OX40L was further incorporated as a candidate immunostimulatory component based on its known role in T‐cell costimulation, although its functional activity within the assembled nanoparticle remains to be directly established. Nanoparticle size critically influences in vivo circulation, cellular uptake, and biodistribution [40]. Previous studies have shown that nanoparticles smaller than 5 nm are rapidly cleared through renal filtration, whereas larger nanoparticles tend to remain at the injection site and are primarily eliminated by local macrophages, thereby limiting their access to draining lymph nodes. In contrast, nanoparticles with a size range of 20–200 nm are considered optimal for dendritic cell uptake and efficient antigen presentation [36, 41, 42].

Nanostructural characterization of recombinant proteins based on the *β*‐annulus peptide using DLS and TEM demonstrated that both *β*‐MEs and OX40L‐*β*‐MEs self‐assembled into spherical nanoparticles with mean diameters of ~25.85 nm and 41.57 nm, respectively, which fall within the optimal size range for lymph node delivery and antigen presentation. Notably, compared with *β*‐MEs nanoparticles, OX40L‐*β*‐MEs nanoparticles exhibited a broader size distribution, which may be related to the relatively large OX40L moiety interfering with precise nanoparticle folding and spatial assembly. Similar phenomena have been observed in other self‐assembling nanoparticle systems displaying large functional proteins or immunomodulatory molecules [43, 44]. Importantly, despite the increased size heterogeneity, OX40L‐*β*‐MEs nanoparticles retained their overall nanostructural integrity, supporting the feasibility of the *β*‐annulus peptide as a vaccine delivery platform.

PRRSV‐specific IgG antibodies were detectable in all vaccinated piglets by 28 dpv and continued to increase thereafter. At 42 dpv, the PRRSV‐specific IgG levels in the *β*‐MEs and OX40L‐*β*‐MEs groups were comparable to those of the inactivated vaccine group, suggesting that *β*‐annulus‐based nanoparticle formulations supported the induction of PRRSV‐specific antibody responses after booster immunization. This discrepancy between detectable PRRSV‐specific IgG and weak neutralizing activity indicates that the antibody responses induced by the current formulations were not predominantly directed toward strongly neutralizing determinants. These findings further underscore the difficulty of inducing potent neutralizing antibody responses against PRRSV and highlight the need for further optimization of antigen selection and conformational presentation to improve the quality of humoral immunity. In addition to humoral immunity, cellular immune responses play a critical role in the control of PRRSV infection. In this study, the OX40L‐*β*‐MEs nanoparticle vaccine not only increased the proportions of CD3^+^, CD4α^+^, and CD8α^+^ lymphocytes but also enhanced lymphocyte proliferation and promoted the secretion of IL‐2, IL‐10, and IFN‐γ. These results suggest that OX40L incorporation may have contributed to the improved cellular immune responses observed in vaccinated piglets. However, antigen‐specific T‐cell functional responses were not comprehensively characterized in the present study. Future studies incorporating additional functional assays, such as ELISpot and broader intracellular cytokine profiling, will be valuable for further defining the magnitude and functional characteristics of vaccine‐induced PRRSV‐specific T‐cell responses. In addition, dendritic cells and other innate immune cell subsets were not phenotypically characterized in the present study. Further investigation of these cell populations may help clarify the mechanisms underlying the immune responses associated with the nanoparticle formulation and OX40L.

During the assessment of vaccine‐mediated protection, piglets in all vaccinated groups showed some degree of disease alleviation following the PRRSV challenge. Piglets immunized with the OX40L‐*β*‐MEs nanoparticle vaccine exhibited only transient fever, followed by recovery to the normal rectal temperature range, and showed significantly higher average daily weight gain than the PBS controls. The OX40L‐*β*‐MEs group also exhibited reduced serum viral loads and milder lung lesions. However, no significant differences were observed between the OX40L‐*β*‐MEs and commercial inactivated vaccine groups in the major protective endpoints evaluated in this study, indicating that the current nanoparticle formulation did not demonstrate superior protective efficacy over the inactivated vaccine. Notably, these protective effects were observed despite the relatively low neutralizing antibody titers induced by the vaccine. These results suggest that nonneutralizing antibody responses, cellular immune‐related responses, or other immune mechanisms may have jointly contributed to the observed protection. Further optimization of the antigen composition, adjuvant formulation, and immunization strategy may help improve the magnitude and durability of vaccine‐mediated protection. Given the genetic diversity and frequent recombination of PRRSV, future studies should evaluate whether this vaccine formulation can provide broader protection against heterologous strains from other circulating lineages.

## 5. Conclusions

In conclusion, this study developed a *β*‐annulus‐based self‐assembling nanoparticle vaccine incorporating selected antigenic regions derived from PRRSV structural proteins and the candidate immunostimulatory component OX40L. The OX40L‐*β*‐MEs nanoparticle vaccine elicited measurable PRRSV‐specific humoral responses and cellular immune‐related responses in piglets. Following an NADC30‐like PRRSV challenge, OX40L‐*β*‐MEs immunization alleviated clinical signs, reduced viral burden, and mitigated lung lesions, indicating partial protection in piglets. These findings support further investigation of the *β*‐annulus‐based nanoparticle strategy for PRRSV vaccine development.

## Author Contributions

Linxing Tian drafted the main manuscript and performed the data analysis. Linxing Tian, Zhengdan Lin, Jiahui Ma, Zihui Hu, Nan Cao, Xinghua Chen, Mingxing Lu, Shudan Liu, Chao Zhang, and Huanchun Chen planned and performed experiments. Xiangmin Li and Ping Qian were responsible for the experimental design.

## Funding

This work was supported by the National Key R&D of China (Grants 2024YFD1800505 and 2023YFD1301902), the Hubei Province Jiangxia Laboratory Open Project (Grant 2025JXKF006), and the National Key Laboratory of Agricultural Microbiology (Grant AML2023B11).

## Ethics Statement

The animal experiments were approved by the Animal Ethical and Welfare Committee of Huazhong Agricultural University, Hubei, China. The animal ethics committee approval number was HZAUSW‐2025‐0094.

## Conflicts of Interest

The authors declare no conflicts of interest.

## Supporting Information

Additional supporting information can be found online in the Supporting Information section.

## Supporting information


**Supporting Information** Figure S1: The proportions of CD3^+^, CD4α^+^, and CD8α^+^ lymphocytes in PBMCs following in vitro PRRSV restimulation were analyzed by flow cytometry at 42 dpv. Table S1: Sequence information of the selected PRRSV antigenic regions in the MEs construct. Table S2: Immunization and challenge schedule for evaluating the protective efficacy of the PRRSV nanoparticle vaccine in piglets. Table S3: Primer sequence information for the RT‐qPCR assay used in this study.

## Data Availability

The data that support the findings of this study are available from the corresponding author upon reasonable request.
