# Supplementary material for: A Self‐Assembling β‐Annulus Nanoparticle Vaccine Incorporating OX40L Confers Partial Protection Against NADC30‐Like PRRSV in Piglets
Source: Transbound Emerg Dis. 2026 Oct 8;2026:2092704. doi: 10.1155/tbed/2092704 (PMC13648024; doi:10.1155/tbed/2092704)
Supplement: Supplementary file 1 [file TBED-2026-2092704-s001.docx]

**A self-assembling *β*-annulus nanoparticle vaccine incorporating OX40L confers partial protection against NADC30-like PRRSV in piglets**

**Linxing Tian ^1,2^, Zhengdan Lin ^1,2^, Jiahui Ma ^1,2^, Zihui Hu^1,2^, Nan Cao ^1,2^, Xinghua Chen ^1,2^, Mingxing Lu ^1,2^, Shudan Liu ^1,2^, Chao Zhang ^1,2^, Huanchun Chen ^1,2,4^, Xiangmin Li ^1,2,3,4*^, and Ping Qian ^1,2,3,4*^**

^1^ National Key Laboratory of Agricultural Microbiology, Hubei Hongshan Laboratory, Huazhong Agricultural University, Wuhan 430070, Hubei, China

^2^ College of Veterinary Medicine, Huazhong Agricultural University, Wuhan, 430070, Hubei, China

^3^ Hubei Jiangxia Laboratory, Wuhan, 430200, Hubei, China

^4^ Key Laboratory of Preventive Veterinary Medicine in Hubei Province, The Cooperative Innovation Center for Sustainable Pig Production, Wuhan, 430070, Hubei, China

**^*^ Correspondence:** lixiangmin@mail.hzau.edu.cn (X. Li); qianp@mail.hzau.edu.cn (P. Qian)

**Supplementary Figure**

 **Supplementary Figure S1.** The proportions of CD3^+^, CD4α^+^, and CD8α^+^ lymphocytes in PBMCs following in vitro PRRSV restimulation were analyzed by flow cytometry at 42 dpv.

**Supplementary Tables**

**Supplementary Table S1**. Sequence information of the selected PRRSV antigenic regions in the MEs construct.

| Component | Source protein | Residues  (start-end) | Length (aa) | Amino acid sequence | Selection rationale |
| --- | --- | --- | --- | --- | --- |
| GP3 | GX-3264 GP3 | 55-99 | 45 | PPCLTRQAAAEIYEPGRSLWCRIGHDRCEESDHDELGFVVPPGLS | Overlaps previously reported antigenic epitopes [1] |
| GP4 | GX-3264 GP4 | 33-70 | 38 | DIKTNTTSASSSVVLQDISCLRHSYPASAAFRKIPQCR | Overlaps a reported antigenically active region [2] |
| GP5-1 | GX-3264 GP5 | 36-60 | 25 | SHLQLIYNLTICELNGTDWLEEKFD | Contains a  neutralization-associated region [3] |
| GP5-2 | GX-3264 GP5 | 130-199 | 70 | CMSWRYSCTRYTNFLLDTKGRLYRWRSPVIIERGGKVDVGGHLIDLKKVVLDGSAATPVTKISAEQWGRP | Contains antibody-reactive and T-cell-associated region [4, 5] |
| M | GX-3264 M | 1-12 | 12 | MGSSIDDFCNLE | Overlaps a previously reported N-terminal antigenic epitope [6] |

Supplementary Table S2. Immunization and challenge schedule for evaluating the protective efficacy of the PRRSV nanoparticle vaccine in piglets.

| Groups | 0 d | 21 d | 42 d |
| --- | --- | --- | --- |
| MEs | 100 μg/2 mL | 100 μg/2 mL | 4 × 10^5^TCID_50_ GX-3264 |
| *β*-MEs | 100 μg/2 mL | 100 μg/2 mL | 4 × 10^5^TCID_50_ GX-3264 |
| OX40L-*β*-MEs | 100 μg/2 mL | 100 μg/2 mL | 4 × 10^5^TCID_50_ GX-3264 |
| PRRSV Inactivated vaccine | 2 mL | 2 mL | 4 × 10^5^TCID_50_ GX-3264 |
| PBS | 2 mL | 2 mL | 4 × 10^5^TCID_50_ GX-3264 |

**Supplementary Table S3**. Primer sequence information for the RT-qPCR assay used in this study.

| Primers | Sequence (5’ to 3’) | Purpose |
| --- | --- | --- |
| ORF6-F | TTGCTAGGCCGCAAGTAC | Detect the viral load of PRRSV |
| ORF6-R | ACGCCGGACGACAAATGC |  |
| ORF6-Probe | FAM-CTGGCCCCTGCCCACCAC-TAMRA |  |

**References**

[1]	Zhou Y-J, An T-Q, He Y-X, Liu J-X, Qiu H-J, Wang Y-F, Tong G (2006) Antigenic structure analysis of glycosylated protein 3 of porcine reproductive and respiratory syndrome virus. Virus Res 118:98–104

[2]	Wang X, Wang Z, Xu H, Biao X, Yang Z (2016) A single amino acid substitution alter antigenicity of glycosylated protein 4 of HP-PRRSV. Virol J 13:129

[3]	Ostrowski M, Galeota JA, Jar AM, Platt KB, Osorio FA, Lopez OJ (2002) Identification of neutralizing and nonneutralizing epitopes in the porcine reproductive and respiratory syndrome virus GP5 ectodomain. J Virol 76:4241–4250

[4]	Rodriguez MJ, Sarraseca J, Fominaya J, Cortés E, Sanz A, Casal JI (2001) Identification of an immunodominant epitope in the C terminus of glycoprotein 5 of porcine reproductive and respiratory syndrome virus. J Gen Virol 82:995–999

[5]	Vashisht K, Goldberg TL, Husmann RJ, Schnitzlein W, Zuckermann FA (2008) Identification of immunodominant T-cell epitopes present in glycoprotein 5 of the north American genotype of porcine reproductive and respiratory syndrome virus. Vaccine 26:4747–4753

[6]	Wang Q, Chen J, Peng J, An T, Leng C, Sun Y, Guo X, Ge X, Tian Z, Yang H (2014) Characterisation of novel linear antigen epitopes on North American-type porcine reproductive and respiratory syndrome virus M protein. Arch Virol 159:3021–3028
